# Path Planning Using a Hybrid Evolutionary Algorithm Based on Tree Structure Encoding

**DOI:** 10.1155/2014/746260

**Published:** 2014-05-28

**Authors:** Ming-Yi Ju, Siao-En Wang, Jian-Horn Guo

**Affiliations:** Department of Computer Science and Information Engineering, National University of Tainan, Tainan 70005, Taiwan

## Abstract

A hybrid evolutionary algorithm using scalable encoding method for path planning is proposed in this paper. The scalable representation is based on binary tree structure encoding. To solve the problem of hybrid genetic algorithm and particle swarm optimization, the “dummy node” is added into the binary trees to deal with the different lengths of representations. The experimental results show that the proposed hybrid method demonstrates using fewer turning points than traditional evolutionary algorithms to generate shorter collision-free paths for mobile robot navigation.

## 1. Introduction 

Path planning is one of the important research issues in mobile robot. When executing a task, the robot is supposed to plan optimal or feasible paths to avoid obstacles in its way and to minimize cost such as time, energy, and distance. Accordingly, path planning can be regarded as an issue in optimization.

A general solution to optimization problem is the use of evolutionary algorithm because of the model-free characteristic. When dealing with an optimization problem, evolutionary algorithm uses some mechanisms inspired by biological evolution to find the approximate solutions. The most famous evolutionary algorithm is genetic algorithm (GA) [1]. Another technology for solving the optimization problem is swarm intelligence. Similarly, the swarm intelligence is inspired from artificial life research. The most famous swarm intelligence is particle swarm optimization (PSO) [2–4]. One of the advantages of GA is its mechanism of probabilistic and useful exchange of information among chromosomes to find an optimal solution, but it is a time-consuming process. In contrast to GA, the advantage of PSO is that each particle's movement is influenced by its local best known position but is also guided towards the best known positions in the search-space to accelerate the convergence. However, PSO does not guarantee that an optimal solution is ever found. Therefore, the idea of integrating two techniques to solve the path planning problem is feasible. Although a number of studies have been made on hybrid GA and PSO, the problem of hybrid algorithms still needs to be improved on the encoding method. Many studies use the fixed number of turning points to represent the path without considering the environmental complexity and then lead to poor path quality. The main reason is due to the solution dimension of PSO which must be fixed. However, the number of turning points used to encode a path should depend on the complexity of the environment. More turning points are needed to accomplish the plan of making a collision-free path in cluttered environments.

This paper adopts a scalable encoding method and develops a novel approach to solve the problem of hybrid GA and PSO. The scalable representation is based on binary tree structure encoding. Each binary tree represents a path. The path can be acquired by tracing the binary tree using the in-order traversal. In the binary tree, each node represents a turn in the path. With more obstacles in the workspace, the binary tree has more nodes. With no obstacles in the workspace, the binary tree only has the starting point and the end point. The number of the nodes depends on the complexity of the environment. The proposed binary tree structure also can solve the learning problem in PSO.

The remainder of this paper is organized as follows.  Section 2 introduces the related work to hybrid evolutionary algorithm.  Section 3 elaborates the proposed hybrid GA-PSO approach as well as a brief explanation of how the binary tree is built.  Section 4 illustrates the experimental results for comparing the proposed hybrid approach with traditional GA and PSO. Concluding remark and future work are presented in Section 5.

## 2. Related Work

The methods for hybrid GA and PSO can be categorized to parallel hybrid [5, 6] and series hybrid [7]. In parallel hybrid strategy, GA and PSO are two independent subsystems. After the evolutionary process starts, the two subsystems execute simultaneously. The main system will check whether the best solution in the two subsystems satisfies the termination criterion or not. If the generations can be divided by the designated iterative times, the main system will perform the hybrid process. In the hybrid process, the main system selects a fixed number of individuals from both subsystems randomly. In contrast, series hybrid strategy uses a series of connections to cascade GA and PSO. In each generation, every individual is sorted according to its fitness value. Only the top-half best-performing individuals, called elites, in each generation are used as parents to generate offspring. The learning mechanism of PSO is adopted to enhance elites. The crossover operator and the mutation operator are then applied to the elites to produce new individuals for the next generation.

We can easily tell the principal differences between these two categories of hybrid methods. Parallel hybrid method generates two different subpopulations by GA and PSO individually for solving a problem. The communication between GA and PSO only exchanges the individuals. In contrast to parallel hybrid, series hybrid uses the same population to search for a better candidate solution iteratively. Series hybrid method applies all the GA and PSO operations on the same population. However, the coding way of all hybrid methods still uses fixed number of turning points to represent a candidate solution for path planning. The required number of turning points in a path should depend on the complexity of the environment. The encoding way using fixed number of turning points is not appropriate. In [8, 9], the traditional grid-based encoding way is used to represent the candidate paths. In contrast to the grid-based encoding, [10] demonstrates a new approach to represent the path based on binary tree structure. Although there are many studies on combining GA and PSO, the main solution to scalable encoding way still remains unsatisfactory.

## 3. Methodology

**Figure 1:**
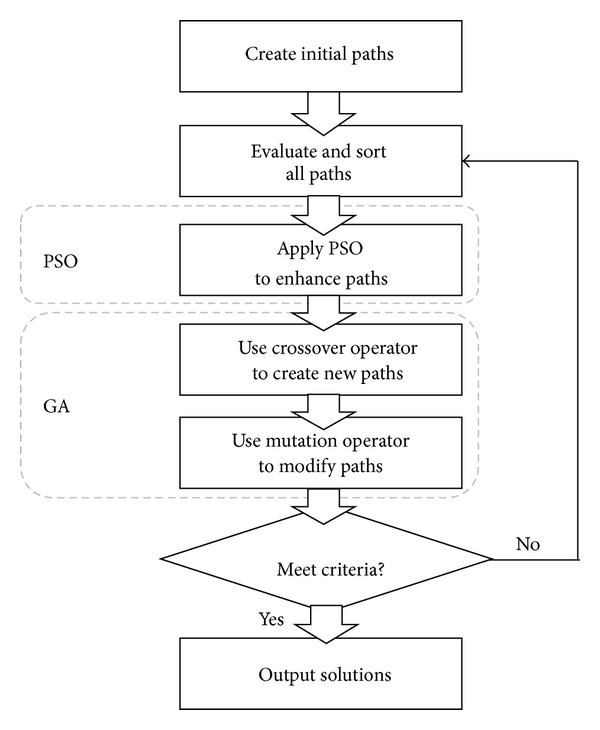
The flowchart of hybrid evolutionary algorithm.

The flowchart of the proposed hybrid evolutionary approach is shown in Figure 1. First, the initial paths and the corresponding binary trees are created. Then, every path is evaluated according to its fitness value. After getting the fitness value, the system sorts the paths from the best to the worst. The top half paths, called elites, are saved and the worst half paths are deleted. The elite paths are enhanced by the PSO operator. Because worst half paths are deleted, the crossover operator is applied to create new paths. When applying the crossover operator, the parents are selected from the elite paths. And each new created path has probability to apply the mutation operator of GA. Finally, the system will check each path to find whether it meets the criteria or not. If no path meets the criteria, the system returns to the evaluating step.

In order to make the hybrid evolutionary algorithm work properly, a good encoding method to combine two kinds of evolutionary algorithms is needed. The proposed hybrid method applies the binary tree structure and adds some dummy nodes to solve the hybrid problem of GA and PSO.

### 3.1. The Creation of Initial Paths

**Figure 2:**
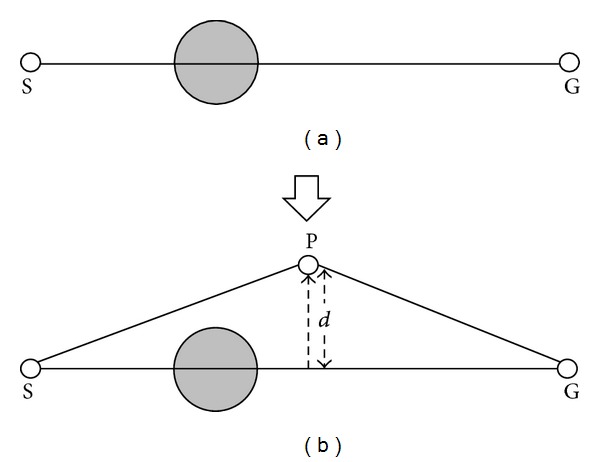
A simple example of creating a collision-free path.

**Figure 3:**
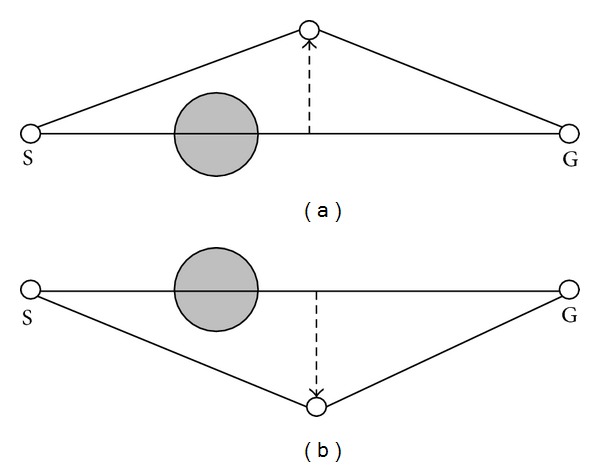
Two directions for a path to create a new turning point.

**Figure 4:**
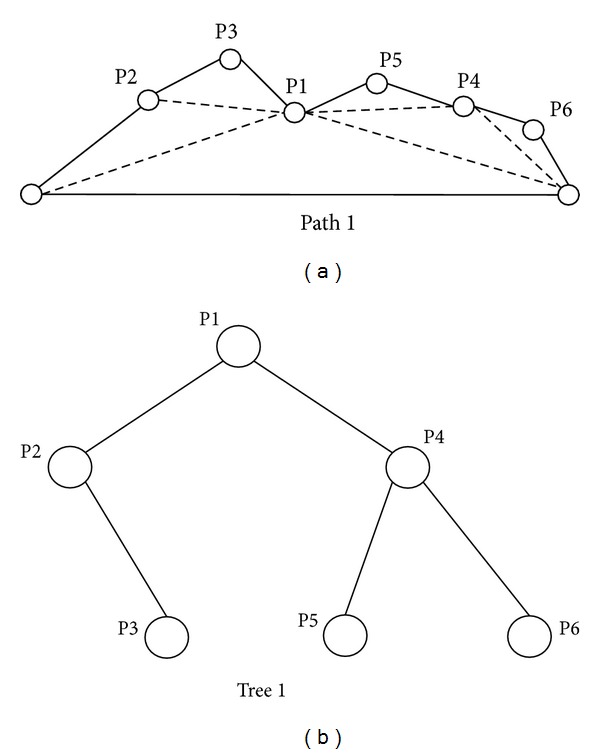
A path with 6 turning points and its corresponding binary tree.

This part describes how to generate a binary tree to represent a path. As shown in Figure 2(a), there is an obstacle between the starting point (S) and the end point (G). First of all, a direct line would be used to connect the starting point and the end point. If there is no obstacle on the line, this line will be the shortest path. If there are obstacles on the line, a new turning point with a random offset distance *d* perpendicular to the line segment would be generated from the middle of the line. An obstacle-free path will be created as shown in Figure 2(b). There are two directions for a line to create a new node, as shown in Figure 3. Every time a new turning point is created, the system will check whether every line segment is obstacle-free or not. If the path is still not obstacle-free, a new turning point will be created until the path can avoid every obstacle. If the number of turning points exceeds the predefined threshold, new turning points will not be created anymore. A path with the maximum number of turning points is not a good candidate solution and will be deleted after the sorting process. As shown in Figure 4, all the turning points of a path are encoded to a binary tree for the process of evolution [10].

### 3.2. Operator of Particle Swarm Optimization

**Figure 5:**
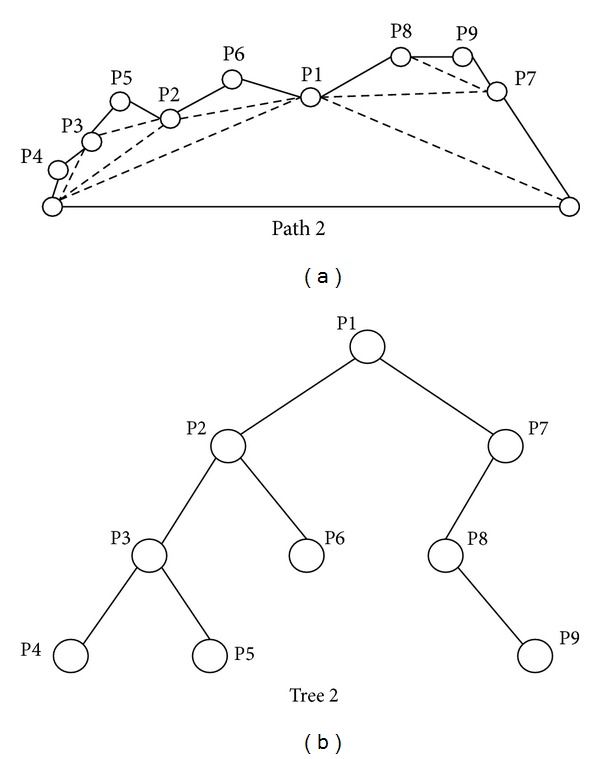
A path with 9 turning points and its corresponding binary tree.

Since PSO does not allow solutions with different dimensions, a novel method is proposed to solve the hybrid problem based on the scalable binary tree structure. Figures 4 and 5 illustrate two different paths and their binary tree representation, respectively. Figures 4(a) and 5(a) show the original paths, and Figures 4(b) and 5(b) represent the corresponding binary trees. Two binary trees have different node numbers and different attitudes. The updating mechanism of PSO cannot apply to two paths with different node numbers. The concept of the proposed hybrid approach is the use of “dummy nodes.” The dummy nodes are created and added when two binary trees have different node numbers in the updating step.

**Figure 6:**
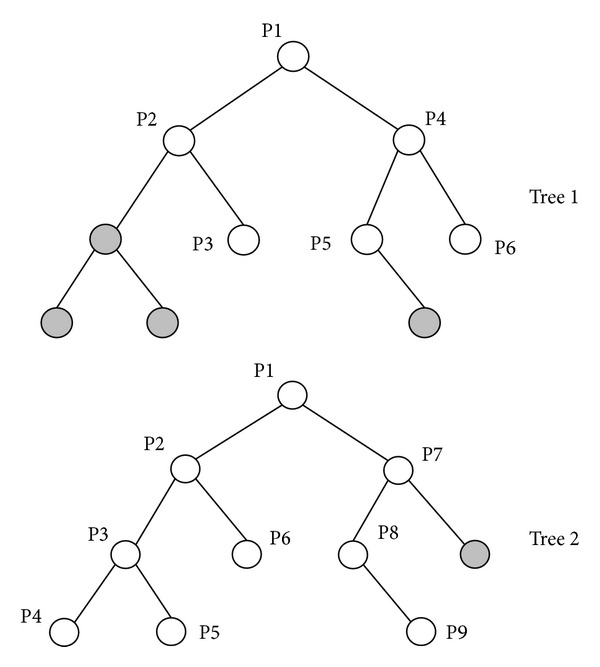
After adding the dummy nodes, symbolized as the grey circles, the two different binary trees have the same attitude.

**Figure 7:**
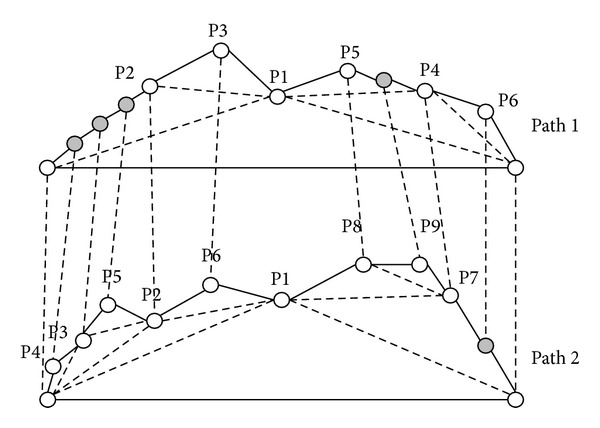
The corresponding path of Figure 6.

In Figure 6, tree 1 has 6 nodes and tree 2 has 9 nodes. Tree 1 will generate four dummy nodes and tree 2 will generate one dummy node, as shown in Figure 6. At this step, tree 1 and tree 2 have the same attitude. The dummy node is different from the common node because the dummy node is a “null” node and the offset distance *d* of dummy node is zero. In the corresponding path of tree 1 and tree 2 as shown in Figure 7, the gray nodes are dummy nodes. According to the updating mechanism of PSO, the path with lower fitness will be influenced by its local best known position but at the same time is guided towards the best known positions in the search space as well. Therefore, the attitude of the path will be similar to the path from which it learns.

Consider that path 1 is the global best solution. As shown in Figure 7, S→ P2 segment in path 1 and S → P4 → P3 → P5 → P2 segment in path 2; the P4, P3, and P5 in path 2 learn from dummy nodes in path 1 so their offset distances will approach zero and the path length can be reduced. After the updating mechanism step, those dummy nodes will be deleted.

### 3.3. Operators of Genetic Algorithm

#### 3.3.1. Crossover Operator

Crossover is a way to produce chromosomes diversity in genetic algorithm. Two individuals can exchange information by applying crossover. In binary tree structure encoding, we exchange the subtree or node to accomplish the crossover operator.

**Figure 8:**
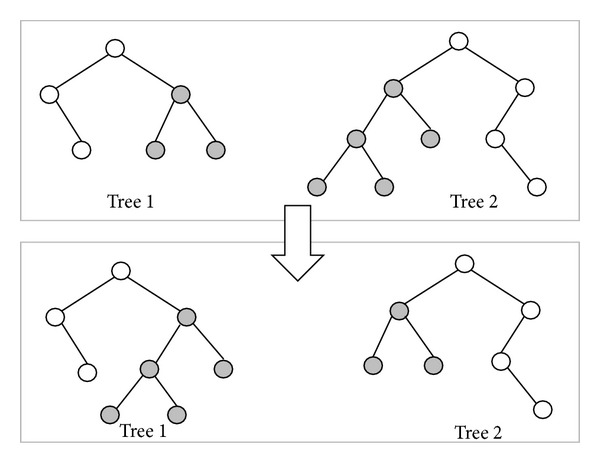
Apply swap subtree crossover operator to generate the offspring from the given parents.

**Figure 9:**
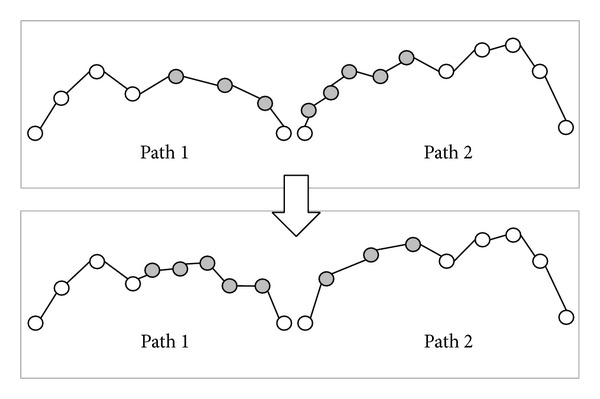
The corresponding paths of Figure 8.

*(a) Swap Subtree Crossover Operator*. First, select two paths randomly. Then, select a node randomly from two paths, respectively. Secondly, exchange two subtrees. The coordinates will be computed again after exchange, in order to update their current correct location. As shown in Figure 8, both tree 1 and tree 2 randomly select subtrees to exchange. The corresponding path applying swap subtree crossover operator is shown in Figure 9.

**Figure 10:**
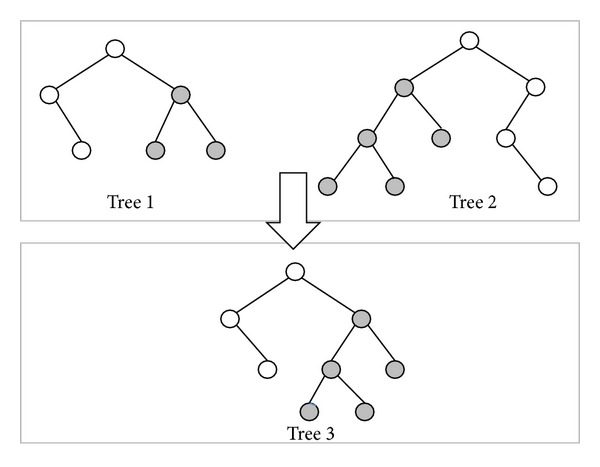
Apply individual crossover operator to generate a new offspring from the given parents.

**Figure 11:**
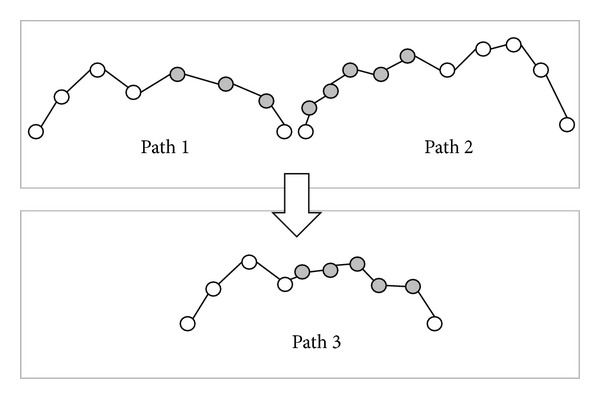
The corresponding paths of Figure 10.

*(b) Generate New Individuals Crossover Operator*. The process is the same as swap subtree crossover operator. First, two paths (tree 1 and tree 2) will be selected randomly. Then, select a node in two trees, respectively. Second, the front part of tree 1 will be combined with the subtree of tree 2 to generate a new tree 3, as shown in Figure 10. And the corresponding path is shown in Figure 11. This operator will produce a new path. Therefore, this operator is used to make up the number of generations.

**Figure 12:**
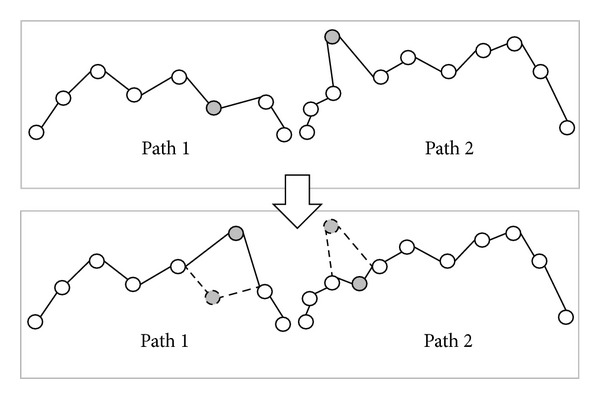
Apply one point swap crossover operator to generate offspring.

**Figure 13:**
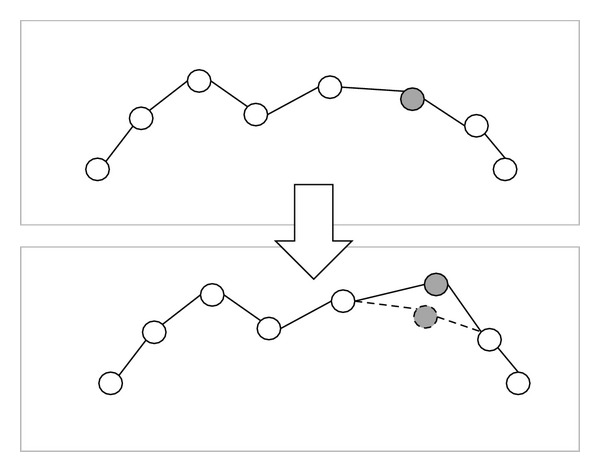
Apply perturb mutation operator to generate offspring.

*(c) One Point Swap Crossover Operator*. The difference between swap subtree crossover operator and one point crossover operator is that the latter only exchanges one node. A simple example is shown in Figure 12 and the corresponding path is shown in Figure 13.

#### 3.3.2. Mutation Operator

Mutation is another way to generate chromosomes diversity in GA. This operator will be applied only when a new path is generated. Mutation changes the contents of a single chromosome so that the path may have a greater variability. Therefore, the system may generate higher probability to find a better solution. Of course, this variation also may make an individual worse. Each node has the possibility to mutate except at start point and end point.

*(a) Perturb Mutation Operator*. The function of perturb mutation is to change the offset distance *d* of a tree node. The new offset distance *d* is generated randomly. A simple example is shown in Figure 13. The gray node becomes farther from the original path than before but the direction does not change.

**Figure 14:**
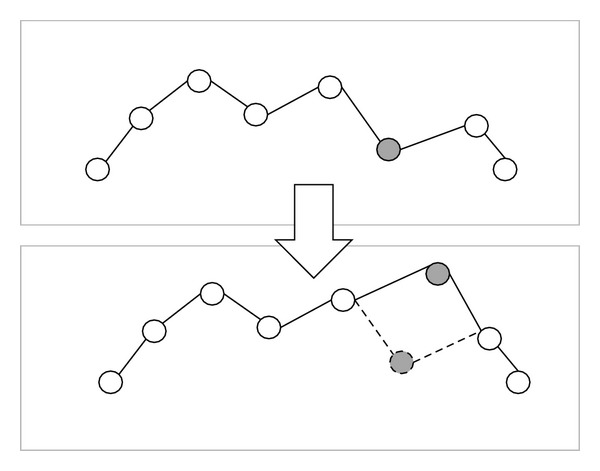
Apply flip mutation operator to generate offspring.

*(b) Flip Mutation Operator*. Flip mutation is similar to the perturb mutation but changes the direction of the offset distance *d*. A simple example is shown in Figure 14. The gray node only change the direction but the distance *d* does not change.

## 4. Experimental Results and Analysis 

In order to evaluate the performance of the proposed hybrid approach, three different types of test environments are used in our experiments.  Table 1 illustrates the control parameters used in those experiments. The number of population size is 30, which represents 30 individuals to perform the evolution. The maximum number of generations is set to 2,000. If the evolution reaches 2,000 generations and does not satisfy the termination threshold, the evolution will be stopped. Mutation probability is set as 0.1%. The inertia weight and learning factors of the PSO are given as 0.5 and 1.3, respectively. The fitness value is defined as the length of a path plus the penalty for collision condition. The penalty is set to 1,000 as collision occurrence; otherwise, the penalty is given as zero. According to different experimental environments, we set the different starting points and ending points.

**Figure 15:**
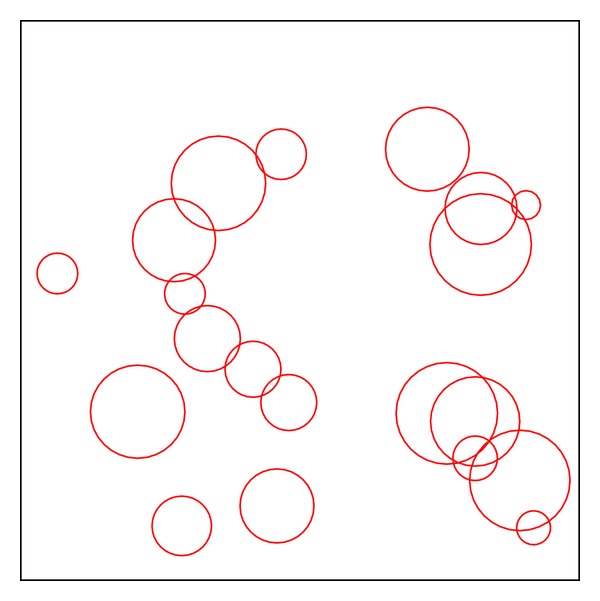
Test environment I.

Figure 15 displays a workspace with 20 overlapped obstacles. In this experiment, the starting point is set at (0, 0) and the ending point at (200, 200). To emphasize genetic length variability of the proposed hybrid method, we use two different tree sizes, 7 and 15 nodes, to perform comparison. Having more nodes can mean having more opportunities to avoid obstacles, but more nodes may also cause unnecessary detour.  Table 2 shows the results for the GA and PSO with 7 tree nodes. According to the results, the proposed hybrid method gets a large advantage on effective path, although the proposed hybrid algorithm loses in the shortest path length of PSO. However, the result indicates that the proposed method is more stable to find a valid path. As the results of GA and PSO with 15 nodes shown in Table 3, the proposed hybrid method and GA won PSO in the valid number of paths.

**Figure 16:**
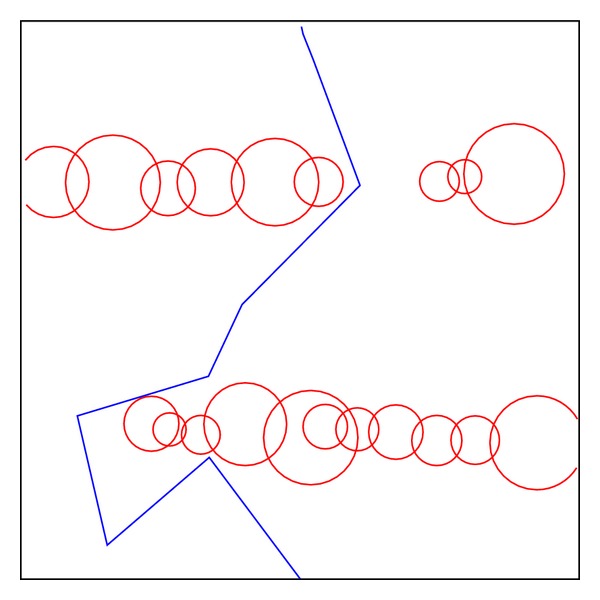
Test environment II.

The second test environment, shown in Figure 16, for the path planning problem is one that is commonly used for performance evaluation. In this form of obstacle distribution, the proposed hybrid method can produce more numbers of effective paths than the other two algorithms. Experimental results are illustrated in Tables 4 and 5. It is noticeable that the number of effective paths generated by the proposed hybrid method is substantially better than GA and PSO.

**Figure 17:**
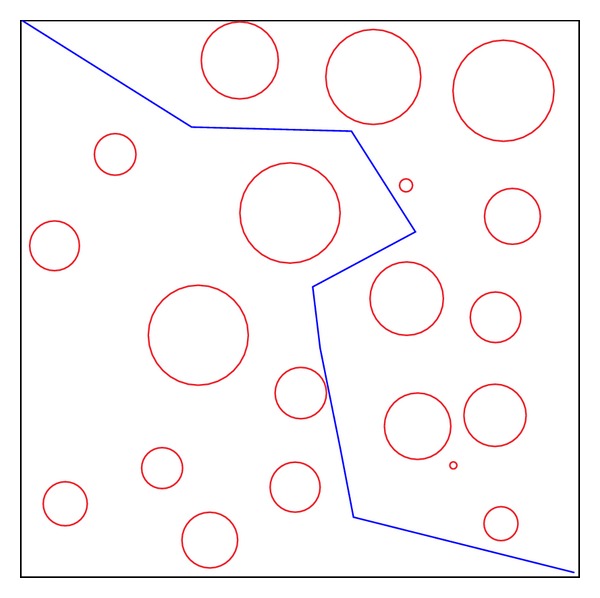
Test environment III.

The difference between the third experiment environment, shown in Figure 17, and the previous ones is that the obstacles do not overlap in the test environment, and so the obstacles take up more proportion on the map. As shown in Table 6, whether in the mean length of effective paths or the number of valid paths, the results of the proposed hybrid method are better than the other two algorithms.

## 5. Conclusion and Future Work

This study describes a method using binary tree structure encoding to hybrid genetic algorithm and particle swarm optimization for solving the path planning problems. Dummy nodes are added to the binary trees to deal with the different lengths of solution/chromosome problem for PSO. The experimental results show that the proposed hybrid method uses fewer turning points than the traditional grid based methods and the number of obstacle-free paths is generated more than traditional evolutionary algorithms. In the future, we will improve this method to generate a curve path so that the method can be applied to the system.

## Figures and Tables

**Table 1:** Experiment parameters.

Name	Value	Comment
Population size	30	
Maximum generation allowed	2000	Termination criterion I
Fitness threshold	10	Termination criterion II: if the change of best fitness is smaller than the predefined threshold by 10 times, terminate the evolutionary process
Crossover probability	1	For GA
Mutation probability	0.001	For GA
Inertia weight	0.5	For PSO
Learning factors	1.3	For PSO

**Table 2:** Comparison in performance among the proposed hybrid approach, PSO, and GA for test environment I.

	Hybrid	PSO	GA
Initial tree size (number of nodes)	≤7	7	7
Number of valid paths	2979	2730	2934
Mean length of valid paths	311.9088	306.2684	314.4024
Standard deviation of valid paths	21.0225	13.9147	21.4342
Shortest valid path	295.5128	295.2754	295.5218
Average solution tree size (average number of nodes)	3.3283	7	7

**Table 3:** Comparison in performance among the proposed hybrid approach, PSO, and GA for test environment I.

	Hybrid	PSO	GA
Initial tree size (number of nodes)	≤15	15	15
Number of valid paths	2996	2931	2996
Mean length of valid paths	314.3656	311.4129	315.1283
Standard deviation of valid paths	21.8718	19.0713	21.4997
Shortest valid path	295.3884	294.8781	295.6429
Average solution tree size (average number of nodes)	3.2774	15	15

**Table 4:** Comparison in performance among the proposed hybrid approach, PSO, and GA for test environment II.

	Hybrid	PSO	GA
Initial tree size (number of nodes)	≤7	7	7
Number of valid paths	2032	307	211
Mean length of valid paths	326.1601	300.8695	316.1638
Standard deviation of valid paths	31.3879	14.8971	17.1472
Shortest valid path	274.7688	283.5653	289.0466
Average solution tree size (average number of nodes)	5.9975	7	7

**Table 5:** Comparison in performance among the proposed hybrid approach, PSO, and GA for test environment II.

	Hybrid	PSO	GA
Initial tree size (number of nodes)	≤15	15	15
Number of valid paths	2620	1410	952
Mean length of valid paths	370.1507	304.9307	320.2028
Standard deviation of valid paths	84.7093	21.1082	20.7493
Shortest valid path	275.2839	270.8929	279.0292
Average solution tree size (average number of nodes)	7.3294	15	15

**Table 6:** Comparison in performance among the proposed hybrid approach, PSO, and GA for test environment III.

	Hybrid	PSO	GA
Initial tree size (number of nodes)	≤15	15	15
Number of valid paths	3000	2982	2966
Mean length of valid paths	317.2784	317.6262	323.0512
Standard deviation of valid paths	22.9993	23.6171	24.3007
Shortest valid path	284.6030	285.6138	286.2925
Average solution tree size (average number of nodes)	4.52167	15	15
